# Metabolic engineering to expand the substrate spectrum of *Pseudomonas putida* toward sucrose

**DOI:** 10.1002/mbo3.473

**Published:** 2017-03-27

**Authors:** Hannes Löwe, Lukas Schmauder, Karina Hobmeier, Andreas Kremling, Katharina Pflüger‐Grau

**Affiliations:** ^1^ Fachgebiet für Systembiotechnologie Fakultät für Maschienenwesen Technische Universität München Garching Germany

**Keywords:** metabolic engineering, *Pseudomonas putida*, sucrose metabolism

## Abstract

Sucrose is an important disaccharide used as a substrate in many industrial applications. It is a major component of molasses, a cheap by‐product of the sugar industry. Unfortunately, not all industrially relevant organisms, among them *Pseudomonas putida,* are capable of metabolizing sucrose. We chose a metabolic engineering approach to circumvent this blockage and equip *P. putida* with the activities necessary to consume sucrose. Therefore, we constructed a pair of broad‐host range mini‐transposons (pSST – sucrose splitting transposon), carrying either *cscA*, encoding an invertase able to split sucrose into glucose and fructose, or additionally *cscB*, encoding a sucrose permease. Introduction of *cscA* was sufficient to convey sucrose consumption and the additional presence of *cscB* had no further effect, though the sucrose permease was built and localized to the membrane. Sucrose was split extracellularly by the activity of the invertase CscA leaking out of the cell. The transposons were also used to confer sucrose consumption to *Cupriavidus necator*. Interestingly, in this strain, CscB acted as a glucose transporter, such that *C. necator* also gained the ability to grow on glucose. Thus, the pSST transposons are functional tools to extend the substrate spectrum of Gram‐negative bacterial strains toward sucrose.

## 1 Introduction

*Pseudomonas putida* is a well‐characterized Gram‐negative soil bacterium endowed with many traits that make it a suitable chassis for contemporary, industrially oriented metabolic engineering. This strain is genetically fully tractable and tolerant toward solvent or oxidative stress (Kim & Park, 2014; Ramos et al., 2015). Therefore, this bacterium is a good candidate for hosting harsh redox reactions, which makes it a favorite workhorse for industrial and environmental biocatalysis. *P. putida* possesses a novel and unique metabolic architecture, the so‐called EDEMP cycle, which enables the bacterium to recycle part of the carbon sources through activities of the Entner‐Doudoroff, the Embden‐Meyerhof‐Parnas, and the pentose phosphate pathways (Nikel, Chavarría, Führer, Sauer, & de Lorenzo, 2015). This ability allows it to adjust NADPH formation, the key cofactor to combat oxidative stress. It is considered a model organism for biodegradation, as it possesses a remarkable capacity to degrade aromatic compounds, such as m‐xylene or toluene (Jiménez, Miñambres, García, & Díaz, 2002) and provides a robust metabolic and biochemical environment, that facilitates recombinant biosynthesis of several valuable natural products, including rhamnolipids, terpenoids, polyketides and nonribosomal peptides (Loeschcke & Thies, 2015). These properties, together with a plethora of SynBio tools (Aparicio, Jensen, Nielsen, de Lorenzo, & Martínez‐García, 2016; Lieder, Nikel, de Lorenzo, & Takors, 2015; Martínez‐García, Aparicio, de Lorenzo, & Nikel, 2014; Martínez‐García, Aparicio, Goñi‐Moreno, Fraile, & de Lorenzo, 2015) and the availability of genome‐wide metabolic models (Belda et al., 2016; Nogales, Palsson, & Thiele, 2008; Puchalka et al., 2008; Sohn, Kim, Park, & Lee, 2010), place *P. putida* on the list of the preferred contemporary SynBio chassis and production platforms (Nikel, Chavarría, Danchin, & de Lorenzo, 2016).

The viability of any biotechnological process depends on the overall costs to convert a certain substrate into a defined product. Even when the bioconversion yield is high, the process can be financially unfavorable, because of high or unstable feedstock costs. Given the importance of cost‐to‐benefit ratios in microbial biotechnology processes, there is a need for organisms that can convert the best‐suited substrate into the desired product. Still, classical substrates, like starch and sucrose from crops, remain the main substrates for fermentation processes, because of high areal yields and easy accessibility for microorganisms. Sucrose is an important disaccharide used in many industrial applications as a substrate as it is a major component of molasses, a cheap by‐product of the sugar industry.

Unfortunately, despite its metabolic diversity, *P. putida* is not able to metabolize sucrose, as transporters and degradation enzymes are missing (Nelson et al., 2002). However, this sugar is an interesting feedstock for industrial applications. Simulations of rhamnolipid production by *P. putida* carrying the genes necessary showed that sucrose and glycerol were superior to glucose with regard to theoretically achievable yields (Wittgens et al., 2011). In order to eliminate this blockage and to broaden the metabolic traits of *P. putida,* we took a metabolic engineering approach to introducing the ability of sucrose uptake and hydrolysis into this strain. Plasmids and plasmid‐based transposons were built that carry the genes *cscA* and *cscB* from the *csc* operon of *Escherichia coli W*. The *cscA* gene encodes a sucrose invertase, which hydrolyzes sucrose‐yielding glucose and fructose, and *cscB* encodes a sucrose permease, which is thought to be responsible for the transport of sucrose into the cell. The engineered plasmids and transposons were then employed to convey the sucrose using phenotype to *P. putida*. To show the potential of the engineered constructs for other Gram‐negative bacteria, they were also used to metabolically engineer *Cupriavidus necator*, formerly known as *Ralstonia eutropha*. Both strains gained the ability to grow with sucrose as the sole carbon source.

## 2 Experimental Procedures

### 2.1 Organisms, strains, and genetic manipulation

The bacterial strains and their sources used in this work are listed in Table S1. *P. putida* eYFP was constructed by the insertion of a constitutively expressed eYFP‐gene via a mini‐Tn7 transposon (Lambertsen, Sternberg, & Molin, 2004) into the genome of *P. putida* KT2440 EM178 (obtained from Víctor de Lorenzo). The oligonucleotides for the construction of plasmids can be found in Table S2. All plasmids and genetic manipulations were performed on *E. coli* DH5α or *E. coli* DH5α λ*‐pir*. Restriction sites and Shine‐Dalgarno sequences (when necessary) were added with the primers during the PCR. Fragments were cloned into the corresponding plasmids with the aid of suitable restriction sites. Plasmids were subsequently transferred to *P. putida* by conjugation with the helper strain *E. coli* HB101 (pRK600) and selected on M9 citrate with suitable antibiotics (de Lorenzo & Timmis, 1994). The pSEVA plasmids and pBAMD1‐2 were used as backbones (Martínez‐García, Calles, Arévalo‐Rodríguez, & de Lorenzo, 2011; Martínez‐García et al., 2015; Silva‐Rocha et al., 2013) and *cscA* and *cscB* were amplified from genomic DNA of *E. coli* W (DSM 1116). For C‐terminal GFP‐tagging of CscB, pVLT_gfp was constructed. Therefore, the GFP‐encoding gene borne by pGREEN plasmid (accession number AB124780) was amplified with PCR primers and cloned into the polylinker of pVLT31 (de Lorenzo, Eltis, Kessler, & Timmis, 1993), originating in pVLT_gfp. The *cscB* gene was amplified by PCR adding a Shine‐Dalgarno sequence, restriction sites and a linker sequence (GSAGSAAGSGEF, (Waldo, Standish, Berendzen, & Terwilliger, 1999)), and cloned upstream of *gfp*, resulting in pVLT_*cscB_gfp*. As control pVLT_gfp_SD (Pflüger‐Grau, Chavarría, & de Lorenzo, 2011) was used.

### 2.2 Cultivation of microorganisms

In order to culture microorganisms for genetic engineering, bacteria were grown in LB medium with an appropriate antibiotic (standard concentrations: 50 mg/L Kanamycin, 200 mg/L Streptomycin, 50 mg/L Spectinomycin, 10 mg/L Gentamycin).

For cultivation studies, all microorganisms were cultivated in three steps unless stated otherwise: Initially, the cultures were cultivated in 5 ml LB medium in culture vials. The second culture step used M9 medium (Miller, 1972) with glucose*,* and MR medium (Lee & Lee, 1996) with fructose for *C. necator* in 100 ml shake flasks filled with 10 ml of medium. The main culture was conducted in 500 ml shake flasks, filled with 50 ml of M9 (*P. putida* or *E. coli*) or MR medium (*C. necator)*, supplied with sucrose as a carbon source. The standard initial substrate concentration was 3 g/L for each sugar. If necessary, a suitable antibiotic (see above) was added. Cultivation temperatures were 30°C for *P. putida* and *C. necator* and 37°C for *E. coli*. Cultures were shaken in an orbital shaker with a rotation frequency of 220 rpm.

For experiments with a large number of conditions to be tested, a cultivation system in a microplate reader (Tecan, Austria) was used. Sterile plates (Greiner, Germany) were filled with 200 μl medium in each well and inoculated with 4.5–5.6 μl of cultures washed in cultivation medium. Optical density (OD) at 600 nm was measured every 20 min after shaking for 60 s over a period of 72 hr. Nonsterile microtiter plates (Nunc, Germany) were used to measure the OD of cultures in shake flasks in the microplate reader (Tecan, Austria) with 200 μl of sample. The growth experiments were performed in three completely independent setups (different days, different precultures originating from different colonies). Additionally, each experiment was done in triplicates, that is, inoculation of three different wells from the same preculture.

### 2.3 Determination and normalization of growth rates in strains with chromosomal inserts

To determine the differences in the growth rates of the individual clones of *P. putida*::miniTn*5‐cscAB,* each experiment was performed three times in triplicates in the microplate reader (Tecan, Austria) as described above. The individual growth rate of each culture of one experiment was determined by linear regression of the logarithmized optical density in the exponential growth phase. The relative growth rate μ/μ_mean_ was determined by calculating the ratio of each growth rate and the mean growth rate of the experiment. Therefore, we compared which strain performs better or worse than average. This was done separately for each experiment. The data shown is the mean and standard deviation of the normalized growth rates of the three independent experiments.

### 2.4 Determination of sugar concentration via HPLC

Sucrose, glucose, and fructose concentrations during fermentation were determined by high performance liquid chromatography (HPLC). Samples were taken from shake flasks and centrifuged for 5 min at 17,000*g*. The supernatant was frozen and stored for analysis. All samples were thawed prior to measuring, filtered with 0.22 μm regenerated cellulose filter plates and injected (20 μl) into the HPLC machine (Agilent 1,100 series). A Shodex SH1011 column was used at a flow rate of 0.5 ml/min with 0.5 mmol/L H_2_SO_4_ as the mobile phase. Every hour, a new sample was injected.

### 2.5 Confocal fluorescence microscopy

Expression of the GFP‐fusion proteins was induced with 0, 0.1 or 1 mmol/L IPTG in LB medium. Cultures were grown overnight with tetracycline and washed in phosphate‐buffered saline. After that, 10 μl of these suspensions were pipetted onto a microscope slide, sealed with a cover slip and directly analyzed by microscopy. For confocal microscopy, an Olympus FluoView 1,000 confocal laser scanning microscope was used with an IX81 inverted microscope stand and a UPLSAPO 60× objective. The laser wavelength was set to 488 nm for optimal excitation of GFP and the emission wave length was considered to be 510 nm. A Kalman Line estimator was applied to reduce noise. The gain of the laser was adjusted for every sample to avoid overexposure of cells that might conceal the distribution of fluorescent protein. Several pictures were taken for each condition and representative images were chosen.

### 2.6 CscA activity assays

To get insight into the mechanism of sucrose cleavage, different fractions (cell extract, supernatant and up‐concentrated cells) of *P. putida* cultures were tested for invertase activity. The experiment was performed in triplicates. Aliquots of *P. putida* PP_0075*::cscA* cultures were harvested by centrifugation for 10 min at 8,000*g* in the exponential growth phase (OD_600_ = 0.81, OD_600_ = 1.03, and OD_600_ = 0.62, respectively). One pellet of each culture was resuspended in 5 mmol/L MgCl_2_ to obtain about 25‐fold up‐concentrated whole cell suspensions. To prepare the cell extracts, pellets of an equal amount of cells (*V* = 1.55 ml, *V* = 1.21 ml, and *V* = 2.0 ml, respectively) were resuspended in an equal volume of 5 mmol/L MgCl_2_, sonicated for 2 min in intervals of 40 s (10 s pulse) with 20% intensity. A quantity of 200 μl of up‐concentrated cells, cell extracts, and 5 mmol/L MgCl_2_ as control were mixed with 1.8 ml of culture supernatant (the native medium) and incubated at 750 rpm in a shaking incubator at 30°C. Sucrose cleavage was followed every hour by taking samples, inactivation for 10 min at 90°C, and subsequent analysis by HPLC. Sucrose depletion was fitted by exponential regression and initial slopes were calculated to obtain the corresponding initial sucrose cleavage rates.

## 3 Results and Discussion

### 3.1 Expression of *cscA* and *cscAB* from plasmids yield sucrose‐utilizing phenotypes in *P. putida*

**Figure 1:**
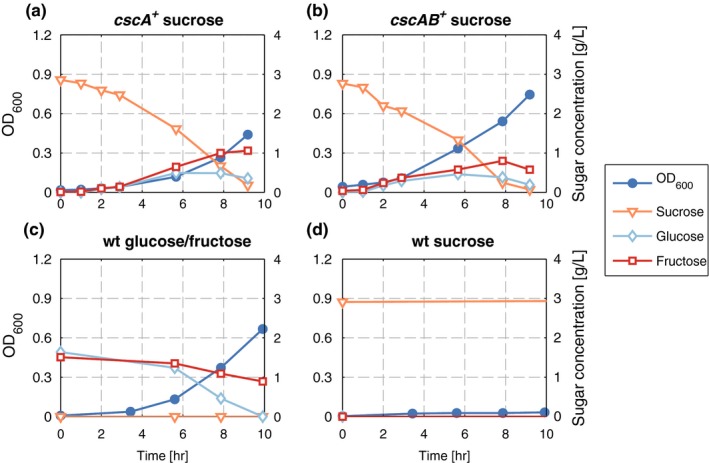
Growth and sugar consumption/production of different *P. putida* strains grown in M9 minimal medium in shake flasks. OD (blue circles), sucrose (orange triangles), glucose (light blue diamonds), and fructose (red squares) were measured in each experiment. (a) *P. putida* (pSEVA224‐*cscA*) with 3 g/L sucrose. (b) *P. putida* (pSEVA224‐*cscAB*) with 3 g/L sucrose (c) *P. putida* eYFP with 1.5 g/L glucose and fructose each (d) *P. putida* eYFP with 3 g/L sucrose

In order to broaden the metabolic traits of *P. putida* and thereby increase its relevance in industrial applications, we set out to construct a strain that is able to metabolize sucrose. We chose *cscA,* encoding an invertase and *cscB*, encoding a sucrose permease from *E. coli W,* as candidate genes. To test their functionality in *P. putida,* either *cscA* or both genes were cloned under the control of an inducible promoter in a plasmid of the pSEVA family (Martínez‐García et al., 2015), resulting in pSEVA224‐*cscA* and pSEVA224‐*cscAB* (Fig. S1). These plasmids were introduced into *P. putida*, and the resulting strains were grown in minimal medium with sucrose as the sole carbon source. *P. putida* carrying either *cscA* or *cscAB* was able to grow on sucrose (Figure 1). Both strains metabolized sucrose, while fructose and glucose were transiently detectable in the culture supernatant until the sucrose was completely split. As control, the plasmid‐free *P. putida* was grown with a mixture of equal parts of glucose and fructose and on sucrose. As expected, it grew well in the presence of the monosaccharides (Figure 1), but showed no growth in the presence of sucrose. Glucose and fructose were metabolized at the same time although glucose uptake was slightly faster than fructose uptake.

Interestingly, *P. putida* carrying either the *cscA* or the *cscAB*‐expressing plasmid did not show any obvious qualitative difference in time courses of sucrose hydrolysis nor in kinetic parameter (Table 1). Growth rate and biomass yield from sucrose were comparable in both cases. The growth rate could not be increased by induction of *cscA* or *cscAB* expression with IPTG. As was the case without IPTG, the sucrose splitting rate was still higher than the uptake of glucose and fructose (data not shown). This indicates that for sucrose consumption by *P. putida,* the expression of *cscB* is dispensable or at least not advantageous. To test this, we performed competition experiments by mixing an equal number of cells of cultures of *P. putida* (pSEVA224‐*cscA*) with cultures of *P. putida* (pSEVA224‐*cscAB*). At the beginning of the experiment and after each transfer, which corresponds to approximately 6.6 generations, we determined the ratio between both plasmids to check whether one was advantageous for growth of the cells. If this were the case, we would expect that clones carrying this plasmid overgrow the others, which would result in a shift of the pSEVA224‐*cscA/*pSEVA224‐*cscAB* ratio toward the advantageous one, or even in complete loss of the less advantageous one. However, we could not observe any trend toward one of the plasmids in the ratio of the plasmid abundance in these competition cultures (Fig. S3). This suggests that CscB production is dispensable for sucrose consumption of the engineered *P. putida*.

**Table 1:** Kinetic growth parameters: growth rate μ, biomass yield from substrate Y_X/S_, for different *P. putida* strains

Genotype	WT	pSEVA224‐*cscA*	pSEVA224‐*cscAB*	PP_0075::*cscA*	PP_3398::*cscAB*
Medium^a^	1.36 g/L Glc + 1.5 g/L Fru	3 g/L Sucrose + 15 mg/L Km	3 g/L Sucrose + 15 mg/L Km	3 g/L Sucrose	3 g/L Sucrose
μ [h^−1^]	0.45 ± 0.02^e^	0.36 ± 0.04^b^	0.38 ± 0.01^b^	0.27 ± 0.05^b^	0.27 ± 0.01^e^
Yxs^d^ [g/g]	0.172 ± 0.009^b^	0.253	0.269	0.210 ± 0.005^b^	0.218 ± 0.003^e^
lag phase [h]^c^	not determined	4.2 ± 1.4	3.4 ± 0.3	not detectable	not detectable

Glc, glucose; Fru, fructose; Km, kanamycin.

a M9 was used as standard medium, only substrates and additives are noted here.

b Standard deviations calculated from three biological replicates.

c Duration of the lag phases were estimated from the intercept of the exponential growth curve with the initial OD_600_, for genomic integrated strains the initial OD was a lot higher which led to no observable lag phase.

d During exponential growth phase.

e Standard deviations calculated from two biological replicates.

But how can the sucrose‐metabolizing phenotype be obtained? There are mainly two plausible explanations: either CscA is acting outside of the cell, or *P. putida* carries porins and transporters that unspecifically facilitate sucrose transport. In the literature, we could not find any indications that *P. putida* possesses transporters that import sucrose. This, of course, does not exclude the presence of a transport protein that aside from its natural sugar is able to transport sucrose as well, maybe to a lower extent. The transient accumulation of glucose and fructose in the culture supernatant independent of the presence of CscB (Figure 1) suggests that sucrose is split outside of the cell. This phenomenon has already been reported for *E. coli* (Kim, Kim, Lee, & Lee, 2013) and was attributed to periplasmic release of CscA into the surrounding medium. Another source for the transient accumulation of glucose and fructose could be an overflow metabolism produced by the rapid intake and hydrolysis of sucrose, especially as the functions are plasmid‐encoded and the proteins should be produced in high amounts due to copy number and induction. One way or the other, the presence of glucose and fructose in the medium could also explain why the additional presence of CscB did not bring any detectable advantage for sucrose‐mediated growth of *P. putida,* as the organism preferably takes up glucose and fructose if present in the medium.

### 3.2 Construction of a broad‐host range shuttle vector and genomic integration of *cscA* and *cscAB* into *P. putida*

**Figure 2:**
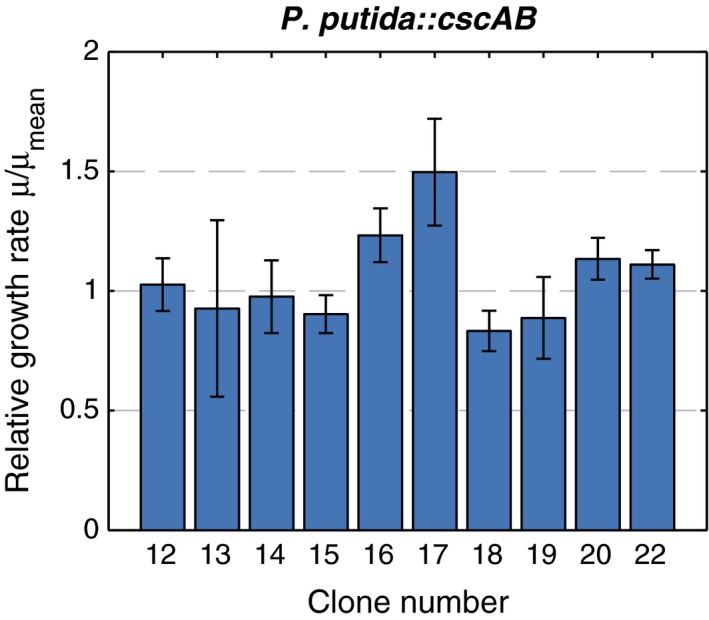
Relative growth rates of different *P. putida* eYFP strains with genomic integration of a polycistronic *cscAB* construct via pSST2. Cells were grown in M9 medium with 3 g/L sucrose in a microplate reader and OD_600_ was measured every 20 min. Growth rates were determined in the log‐linear parts of the growth curves and normalized to the respective mean growth rate of each experiment. Standard deviations were calculated from these normalized growth rates of three independent experiments

As a next step, we introduced *cscA* and *cscAB* into the chromosome of *P. putida*. This was done on one hand to reduce the metabolic burden of plasmid replication and eliminate the need for antibiotics. On the other hand, after genomic integration, *cscA* and *cscB* are present in a single copy, which should reduce the probability for a potential overflow of certain metabolites due to high amounts of the heterologous proteins. For genomic integration, we constructed a broad‐host range shuttle vector, based on the pBAMD1‐2 mini‐Tn*5* transposon vector (Martínez‐García et al., 2011), containing either *cscA* alone or the *cscAB* cassette (Fig. S1). Both cassettes were placed under transcriptional control of the P_*trc*_ promoter devoid of the LacIq repressor binding site, in order to obtain constitutive transcription. With the resulting plasmids pSST1 (sucrose splitting transposon 1 with *cscA*) and pSST2 (sucrose splitting transposon 2 with *cscAB*), we successfully integrated the genes randomly into the chromosome of *P. putida*. To check whether the integration site, that is, disruption of the gene where the cassette has inserted, has an influence on sucrose consumption, we chose 22 transformants from the genomic integration of the Tn*5*‐*cscAB* cassette of pSST2 for a more detailed analysis. Ten of them showed reproducible growth in M9 minimal medium with sucrose. These were compared in parallel growth experiments in a microplate reader to determine the growth rate of each construct (Figure 2). The experiment was performed three times, each in triplicates. We observed that the absolute values for the growth rates varied between different experiments, but the relative differences were conserved. Therefore, growth rates were normalized by the mean growth rate of all single clones in each round of experiments in order to compare the results from all experiments (Figure 2). Clone #17 grew slightly faster in each round of experiments compared to the other clones, which is reflected by the highest relative growth rate (Figure 2).

Next, we mapped the integration site of the Tn*5*‐*cscAB* cassette. As expected, the mini‐Tn*5* integrated randomly along the chromosome. We could not draw any conclusion regarding the general fitness of the different clones from the gene annotations of the integration sites (Table 2, Fig. S2). Therefore, we decided to use clone #17 for all further studies, in which the gene cassette has inserted into PP_3398, annotated as Curlin‐associated repeat protein. We also screened various clones with the integrated Tn*5*‐*cscA* cassette. As with the Tn5‐*cscAB* cassette, no remarkable difference was observed (data not shown) and we chose the clone with the highest growth rate for further analysis, in which the cassette had inserted into PP_0075, annotated as sulfate transporter.

**Table 2:** Integration of pSST2 in *P. putida* eYFP and mapping of insertion sites

Clone	Gene(s)	Genetic Locus/Loci
12	Hypothetical protein/ISPpu13, transposase Orf3	PP_3983/PP_3984
13	Acriflavine resistance protein/transcriptional regulator, GntR family	PP_2065/PP_2066
14	Alcohol dehydrogenase, zinc‐containing	PP_1720
15	Nitrate‐binding protein NasS, putative/dihydroorotate dehydrogenase	PP_2094/PP_2095
16	DNA‐binding response regulator GltR/sensor histidine kinase	PP_1012/PP_1013
17	Curli fiber major subunit CsgA	PP_3398
18	Sensory box histidine kinase/response regulator; transcriptional regulator, GntR family	PP_3544/PP_3545
19	ATP‐dependent Clp protease, ATP‐binding subunit ClpA	PP_4008
20	S50 ribosomal protein L19/long‐chain acyl‐CoA thioester hydrolase family protein	PP_1465/PP_1466
22	Polyamine ABC transporter substrate‐binding protein	PP_2195

**Figure 3:**
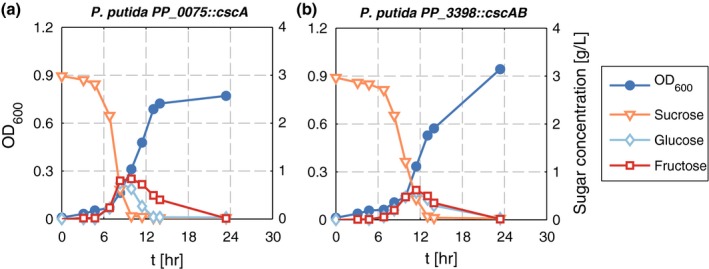
Growth and sucrose consumption by (a) *P. putida* PP_0075*::cscA* and (b) *P. putida* PP_3398*::cscAB*. Cells were grown in M9 minimal medium in shake flasks with 3 g/L sucrose. OD (blue circles), sucrose (orange triangles), glucose (light blue diamonds), and fructose (red squares) were measured in each experiment. Shown are representative curves of three independent experiments

Next, both strains, *P. putida* PP_0075*::cscA* and *P. putida* PP_3398*::cscAB,* were analyzed in more detail during growth on sucrose. Both strains showed almost equal growth kinetics with almost identical growth rates and very similar biomass yields from sucrose (Table 1). In both cultures, sucrose was hydrolyzed and glucose and fructose accumulated transiently (Figure 3). The strains with the chromosomal insertion of *cscA* or *cscAB* had about 30% reduced growth rates compared to the plasmid‐based systems. The reduced growth rates are a consequence of the lower sucrose hydrolysis, which is reflected by a slower decrease in sucrose compared to the plasmid‐bearing cultures. This probably has its origin in different expression levels of the genes due to different promoters and gene copy numbers. As with the plasmid‐based strains, a transient accumulation of glucose and fructose was observed. Both sugars started to be detectable in the culture medium when the cells started to grow, and accumulated to concentrations of 0.5 to 1 g/L. With increasing cell number, glucose and fructose disappeared from the medium, as they were taken up and metabolized. These data hint toward the extracellular splitting of sucrose by a fraction of CscA leaking out of the cells and make the accumulation of glucose and fructose as a result of overflow metabolism unlikely.

Still, as with the plasmid‐based strains, no difference could be observed between the strain expressing only the invertase and the one additionally producing the sucrose permease. Thus, even at a lower sucrose splitting rate, no significant effect of the presence of *cscB* in the genome could be observed. This raised the question of whether the sucrose permease is functionally produced at all in *P. putida*.

### 3.3 CscB is produced and localizes to the cell membrane

As the presence of *cscB* encoding the sucrose permease did not lead to any detectable effect when *P. putida* cells were grown on sucrose, we aimed to show that it is indeed expressed and to compare its production and localization in *P. putida* to the one in *E. coli* where it was shown to be functional (Sahin‐Tóth, Frillingos, Lengeler, & Kaback, 1995).

**Figure 4:**
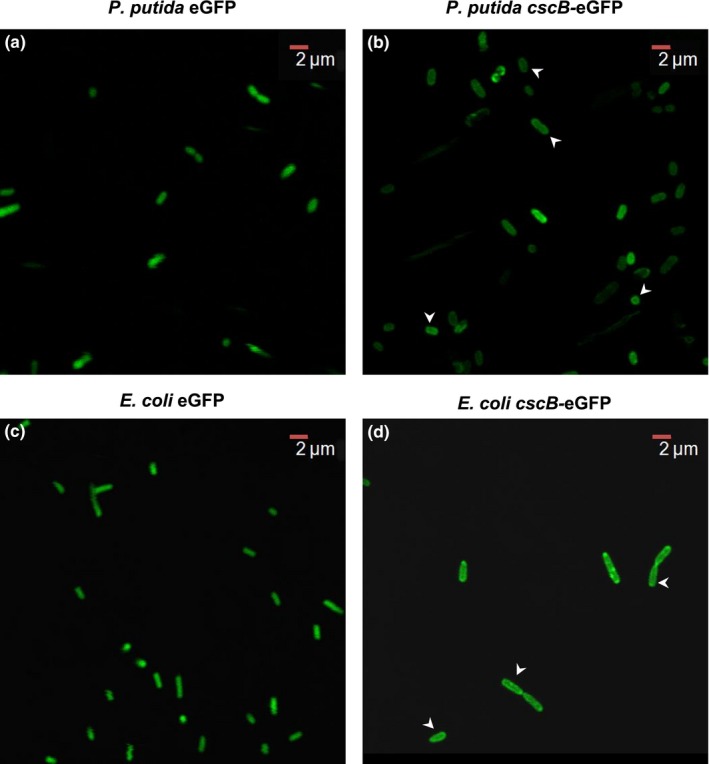
CscB production and localization in *P. putida* and *E. coli*. Confocal fluorescence microscopy of (a) *P. putida* (pVLT_gfp), (b) *P. putida* (pVLT_*cscB*‐GFP), (c) *E. coli* (pVLT_*gfp*), and (d) *E. coli* (pVLT_*cscB*‐GFP). Pictures were taken with an Olympus FluoView 1000. Arrows point to the cells where the membrane localization rings are most clear

Therefore, we expressed it as an N‐terminal fusion to GFP from an inducible plasmid in *P. putida* and in *E. coli*. As control, GFP alone was produced from the same plasmid in both strains. The cellular location of the fluorophore was examined by confocal microscopy. As expected, in the controls, the fluorescent signal is evenly distributed in the cytoplasm (Figure 4a, c). In *P. putida* as well as in *E. coli* expressing a GFP‐*cscB* fusion protein, another distribution of the fluorescent signal is observed (Figure 4b, d). In both organisms, a uniformly fluorescing ring surrounding the cell can be seen, which hints toward a localization of the CscB protein to the membrane of both organisms. Additionally, foci with higher fluorescence occur, which might well be inclusion bodies or unspecific aggregates (Landgraf, Okumus, Chien, & Baker, 2012). Thus, we can assume that the CscB protein itself is produced and behaves in a similar fashion as in *E. coli*. However, these data do not explain why the production of the sucrose permease has no effect on growth of *P. putida* on sucrose.

### 3.4 CscB acts as glucose transporter in *C. necator*

**Figure 5:**
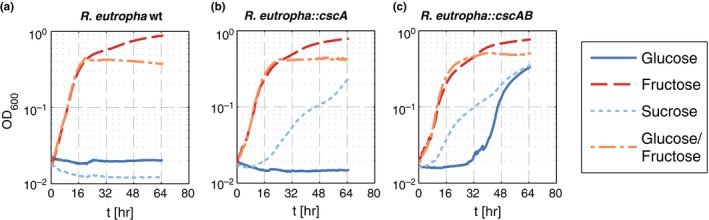
Growth of *C. necator* wt (a), *C. necator*::miniTn*5*‐*cscA* (b), and *C. necator*::miniTn*5*‐*cscAB* (c) with either fructose (red dashed line), sucrose (light blue dotted line), or glucose (solid dark blue line), or glucose/fructose (orange dotted and dashed line) as the single carbon source. Note that the presence of CscA is sufficient to allow growth on sucrose, whereas the expression of *cscB* additionally allows growth on glucose

To show the broad‐host range potential of the constructed transposon and the ability to transfer sucrose metabolism to bacteria other than *P. putida*, we chose *C. necator* as the target. *C. necator* is already used industrially in the production of poly‐3‐hydroxybutyrate (PHB) (Chen, 2009), but the selected strain, *C. necator* H16, is not naturally able to metabolize glucose, sucrose or any other sugar except for fructose and N‐acetylglucosamine (Pohlmann et al., 2006; Sichwart, Hetzler, Bröker, & Steinbüchel, 2011). The vectors pSST1 and pSST2 were transferred from *E. coli* to *C. necator* via triparental mating and the transposon‐carrying cells were identified by antibiotic selection (Km^R^). Both strains, *C. necator*::miniTn*5*‐*cscA* as well as *C. necator*::miniTn*5*‐*cscAB,* were able to grow on sucrose as the sole carbon source, whereas the wild type was not (Figure 5). Thus, the pSST plasmids could be used successfully to bring about the ability to split sucrose not only to *P. putida* but also to *C. necator*. Whether sucrose is extracellularly cleaved by leaking invertase and cells are growing on fructose or whether sucrose is transported into the cell by the activity of CscB cannot be deduced from the data. However, we could show that CscB acts as glucose transporter in this strain. In the growth experiments, the *C. necator* strains were also cultivated with fructose as positive control and glucose as negative control. Surprisingly, *C. necator*::mini‐Tn*5‐cscAB* was able to metabolize glucose (Figure 5c), whereas *C. necator*::mini‐Tn*5‐cscA* was not (Figure 5b). The organism is equipped with everything needed for growth on glucose except for transport activity. When a suitable glucose transporter is heterologously expressed, growth is possible (Sichwart et al., 2011). To the best of our knowledge, it has not been shown before that CscB can act as a glucose transporter – in fact (Sugihara, Smirnova, Kasho, & Kaback, 2011) stated that CscB catalyzes the transport of sucrose, fructose and lactulose, but shows no recognition of glycopyranosides, in particular glucose, as this sugar was not able to inhibit sucrose transport in *E. coli*. From our experiments, transport activities other than glucose can neither be confirmed nor excluded. Nevertheless, the functionality of the sucrose permease CscB in *C. necator* shows that, in principle, CscB is transcribed and translated into a functional protein – at least in *C. necator*. However, this does not explain the missing activity for sucrose transport of CscB in *P. putida*.

### 3.5 CscA activity is mainly intracellular

**Figure 6:**
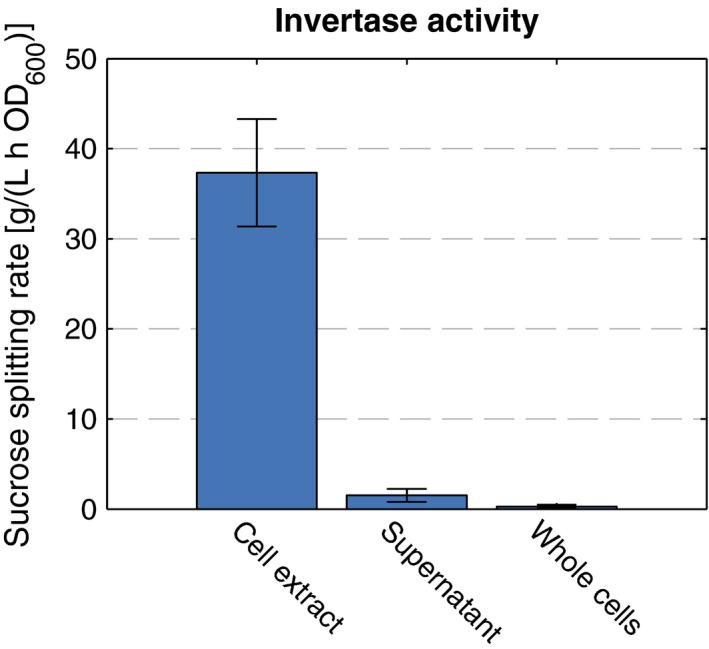
CscA activity of *P. putida* PP_0075*::cscA*. Sucrose cleavage rates were determined from the cell extracts, the culture supernatants, and the whole cells of *P. putida* PP_0075*::cscA*. The mean activity of the supernatants is shown in relation to the optical density at the time of harvest, mean activity of the whole cells and cell extracts in relation to the optical density of the cells used for the assay. The mean values and the corresponding standard deviations of three independent experiments are depicted

A possible scenario for explaining the similar phenotype of strains producing just the invertase CscA or additionally the permease CscB, is a high extracellular sucrose splitting rate of CscA. In this case, sucrose would be hydrolyzed fast enough outside of the cell to make CscB superfluous. This implies that CscA should either be associated with the membrane or occur extracellularly. However, at least in its native host *E. coli* W, it is a cytoplasmic protein, cleaving sucrose intracellularly (Sabri, Nielsen, & Vickers, 2012). To test this scenario and to localize the main activity of CscA, we determined the sucrose cleavage rates of *P. putida* PP_0075*::cscA* and *P. putida* PP_3398*::cscAB* in the culture, the culture supernatant, and in cell extracts (Figure 6). In either strain, the vast majority of activity (>90%) could be found intracellularly and only a small fraction was detected in the culture supernatant. Another, even smaller fraction seems to be associated with the cell, as cleavage rates were marginally higher in the culture compared to the supernatant. We assume that the extracellular activity is a result of nonspecific leaking of CscA out of the cell or of freed CscA after cell lysis. Interestingly, this small fraction of CscA seems to be sufficient for the sucrose‐metabolizing phenotype of *P. putida*. This is a good starting point for further optimization of the sucrose‐metabolizing *P. putida* strains, which is currently in progress in our laboratory.

## 4 Conclusion

With the pSST transposon‐mediated integration of the *csc* genes conferring sucrose metabolism, the first step toward a sucrose‐consuming *P. putida* was undertaken. This opens a new field of potential applications for this otherwise remarkably versatile organism by adding sucrose to the repertoire of substrates of *P. putida*. Further attempts will be aimed at increasing the growth rate either by introducing a functional sucrose transporter into the membrane for efficient sucrose uptake and thus exploitation of the full potential of the intracellular the invertase activity, or by exporting CscA out of the cell for extracellular cleavage. We expect to reach growth rates equal to those observed with glucose and fructose in either case as the cleavage rate of CscA is not the liming step, but the access to sucrose is. The pSST transposons can be used to transfer the sucrose‐consuming phenotype also to other Gram‐negative organisms as well, which was demonstrated here with *C. necator*. However, the functionality of the CscB permease remains somewhat ambiguous: although in *P. putida*, no effect could be assigned to the presence of the protein, in *C. necator* there is a strong indication that it does not only transport sucrose but also glucose across the membrane.

## Conflict of Interest

The authors declare no conflict of interest.
