# Supplementary material for: Metabolic engineering to expand the substrate spectrum of Pseudomonas putida toward sucrose
Source: Microbiologyopen. 2017 Mar 27;6(4):e00473. doi: 10.1002/mbo3.473 (PMC5552902; doi:10.1002/mbo3.473)
Supplement: Supplementary file 1 [file MBO3-6-na-s001.pdf]

SUPPORTING INFORMATION

for

Metabolic engineering to expand the substrate spectrum of *Pseudomonas putida* towards sucrose

by

*Hannes Löwe, Lukas Schmauder, Karina Hobmeier, Andreas Kremling, and Katharina Pflüger-Grau **

Fachgebiet für Systembiotechnologie, Fakultät für Maschienenwesen, Technische Universität München, 85748 Garching (Germany)

Short title: Sucrose-consuming *Pseudomonas putida*

* For correspondence: Katharina Pflüger-Grau, Fachgebiet für Systembiotechnologie, Technische Universität München, Boltzmannstr. 15, 85748 Garching, Germany, Tel.: +49 89 289 15765; Fax.: +49 89 289 15766, Email: k.pflueger-grau@tum.de

This file contains:

Supplementary tables

Table S1: Bacterial strains that were used in this work……………………………………………3
Table S2: Oligonucleotides used for cloning in this study……………………………………4
Table S3: Plasmids used and constructed in this work……………………………………...5
Table S4: Growth rates of individual clones of *P. putida::cscAB*……………………………..6

Supplementary figures

Figure S1: Schematic map of the vectors and plasmids ……………………………………7
Figure S2: Localization of *cscAB* integration along the chromosome of *P. putida* ………...8
Figure S3: Competition of *P. putida* (pSEVA224-*cscA*) and *P. putida* (pSEVA224-*cscAB*) during growth on sucrose……………………………………………………………9

References……………………………………………………………………………………10

Table S1 | Bacterial strains that were used in this work

Bacterial strain	Genotype	Function	Reference
E. coli DH5***α***	F⁻ ***λ***⁻ *endA*1 *glnX44*(AS) *thiE1 recA1 relA1 spoT1 gyrA96*(Nal^R) *rfbC1 deoR nupG* ***Φ***80(*lacZ**Δ**M15*) ***Δ***(*argF-lac*)U169 *hsdR17*(r_K^- m_K^+)	Cloning host	Grant *et al.*, 1990
E. coli DH5***α λ****pir*	F⁻ ***λ*** ⁻ *endA*1 *glnX44*(AS) *thiE1 recA1 relA1 spoT1 gyrA96*(Nal^R) *rfbC1 deoR nupG* ***Φ***80(*lacZ**Δ***M15) ***Δ***(*argF-lac*)U169 *hsdR17*(r_K^- m_K^+), ***λ*** *pir* lysogen	Cloning host	Laboratory collection
R. eutropha H16	wild-type	Expression host; production strain	Wilde, 1962
P. putida KT2440 EM178 *att::YFP-Gm*	Derivative of KT2440; ***Δ***PP3849-PP3920 (prophage 1) ***Δ***PP3026-PP3066 (prophage 2) ***Δ***PP2266-PP2297 (prophage 3) ***Δ***PP1532-PP1586 (prophage 4); constitutively expressed eYFP inserted into *att*-site via a Mini-Tn7 transposon	Expression host; production strain	This work
E. coli HB101	F⁻ ***λ***⁻ h*sdS20*(r_B^- m_B^-) *recA13 leuB6*(Am) *araC14* Δ(*gpt-proA*)62 *lacY1 galK2*(Oc) *xyl-5 mtl-1 thiE1 rpsL20*(Sm^R) *glnX44*(AS)	Helper strain for conjugation	Boyer and Roulland-Dussoix, 1969
E. coli W (DSM 1116)	Wild type	Source of genes	Waksman and Reilly, 1945

Table S2 | Oligonucleotides used for cloning in this study with name, sequence and function. Start codons are underlined, restrictions sites are in italics, and Shine-Dalgarno sequences are presented in bold.

Name	Sequence	Function
cscB_SD_fw	TTTT*GGATCC***AGGAGG**CTTCATATGGCACTGAATATTCC	Amplification of *cscB*
rvPrimer_cscB	TTTT*GGATCC*ATGGCACTGAATATTCC	
cscA_SD_fw	TTTT*GAATTC***AGGAGG**CTTCATATGACGCAATCTCG	Amplification of *cscA*
rvPrimer_cscA	AAAA*GGATCC*TTAACCCAGTAGCCAGAG	
fwP_cscAB_SacI	TTTT*GAGCTC*TTGACAATTAATCATCCGGCTCGTATAATGTGTGGCAATTTCACACCCTAGG	Cloning of a polycistronic *cscAB* construct into pBAMD1-2 with a constitutive promoter
rvP_cscAB_PstI	AAAA*CTGCAG*CTATATTGCTGAAGGTACAGGCGTTTCCATAACTATTTGC	
GFPt(XmaI)_for	TTTTTT*CCCGGG*ATGAGTAAAGGAGAAGAACTTTTCA	Construction of pVLT_*gfp*
fwP_cscB_SD_Eco2	TTTT*GAATTC***AGGAGG**CTTCATATGGCACTGAATATTCC	Construction of a *cscB*-*gfp* fusion protein with pVLT_gfp_SD
rvP_cscB_L_SacI	AAAA*GAGCTC*GAGAATTCGCCACTTCCAGCCGCACTACCTGCACTGCCTATTGCTGAAGGTACAG	
rvP_seq_pVLTgfp	GAATTGGGACAACTCCAGTG	Sequencing of the *cscB*-gfp fusion protein on pVLT_*cscB_gfp*

Table S3 | Plasmids used and constructed in this work

Plasmid name	Description	Source or reference
pSEVA224	Cloning vector; R2K-ori; Km-resistance; IPTG-inducible P_{trc}-promoter	Silva-Rocha *et al.*, 2013
pSEVA434	Cloning vector; pBBR1-ori; Sm/Sp-resistance; IPTG-inducible P_{trc} -promoter	Silva-Rocha *et al.*, 2013
pBAMD1-2	Mini-Tn*5* cloning vector for random chromosomal integration of DNA within mosaic sites; backbone-resistance: Ap; insert resistance: Km	Martínez-García *et al.*, 2014
pSEVA224-*cscA*	Derivative of pSEVA224; *cscA* gene from *E. coli W* integrated into the MCS with EcoRI and BamHI	This work
pSEVA434-*cscB*	Derivative of pSEVA434; *cscB* gene from *E. coli W* integrated into the MCS with BamHI and HindIII	This work
pSEVA224-*cscAB*	Derivative of pSEVA224-cscA; *cscB* gene from *E. coli W* integrated into the MCS with BamHI and HindIII	This work
pBAMD1-2-*cscA*	Derivative of pBAMD1-2; *cscA* gene from pSEVA224-*cscA* integrated into the MCS with SacI and PstI	This work
pBAMD1-2-*cscAB*	Derivative of pBAMD1-2; *cscAB* gene from pSEVA224-*cscAB* integrated into the MCS with SacI and PstI	This work
pRK600	Helper plasmid for conjugation; ColE1 *ori*; Cm-resistance; RK2 (*mob*$^+$ *tra*$^+$)	Kessler *et al.*, 1992
pVLT_*gfp*	Derivative of pVLT31; GFP was cloned into the multiple cloning site with the restriction enzymes XmaI and HindIII	Pflüger-Grau, unpublished
pVLT_*gfp*_SD	Derivative of pVLT31; GFP was cloned into the multiple cloning site with the restriction enzymes SacI and HindIII with start codon and a suitable ribosome binding site	Pflüger-Grau *et al.*, 2011
pVLT_*cscB*-*gfp*	Derivative of pVLT_*gfp*; *cscB* was cloned in front of GFP with a peptide linker (GSAGSAAGSGEFSSSVPG) with the restriction enzymes EcoRI and SacI	This work

Table S4 | Growth rates of individual clones of *P. putida::cscAB.* Shown are the growth rates of three independent experiments. The numbers are the mean and standard deviation of one experiment performed in triplicates. Note that the relative differences between the growth rates of the individual clones are conserved.

**Clone**		**Growth rate μ [h^{-1}]**	
	Experiment 1	Experiment 2	Experiment 3
#12	0.376±0.018	0.15±0.02	0.245±0.009
#13	0.39±0.03	0.17±0.03	0.122±0.005
#14	0.37±0.05	0.14±0.04	0.208±0.003
#15	0.29±0.05	0.13±0.04	0.237±0.008
#16	0.395±0.004	0.22±0.07	0.275±0.009
#17	0.51±0.02	0.27±0.03	0.30±0.02
#18	0.29±0.07	0.12±0.03	0.210±0.009
#19	0.23±0.02	0.173±0.016	0.215±0.014
#20	0.357±0.019	0.18±0.04	0.299±0.015
#22	0.38±0.06	0.17±0.05	0.273±0.017

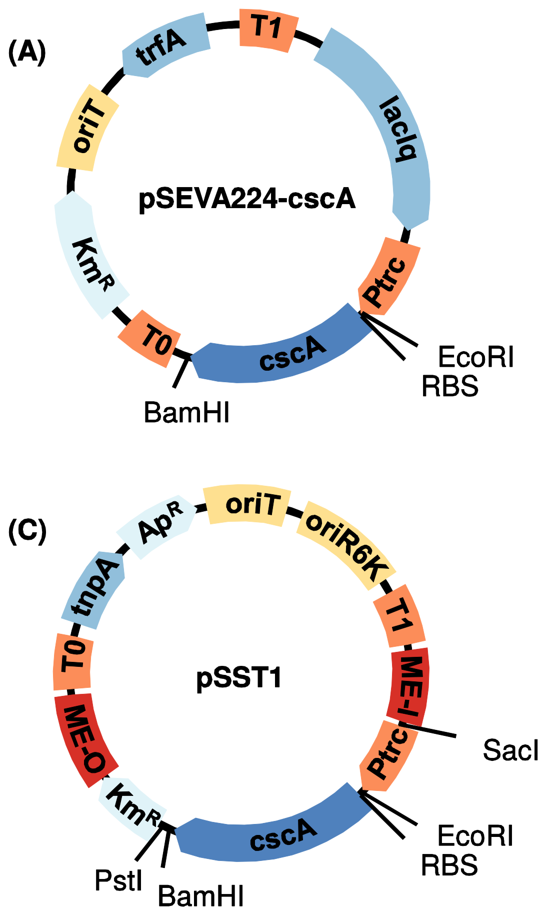

Figure S1 | Schematic map of the vectors and plasmids constructed in this study. The two upper vectors (A and B) are derivatives of pSEVA224. The gene *cscA* or a polycistronic *cscAB* construct containing genes from the *csc* operon from *E. coli* W were amplified via the primers specified in Table S2. Vector and insert were cut via suitable restriction enzymes and ligated. The lower two vectors (C and D) show the same insert transferred to the (identical) multiple cloning site of the mini-transposon vector pBAMD1-2.

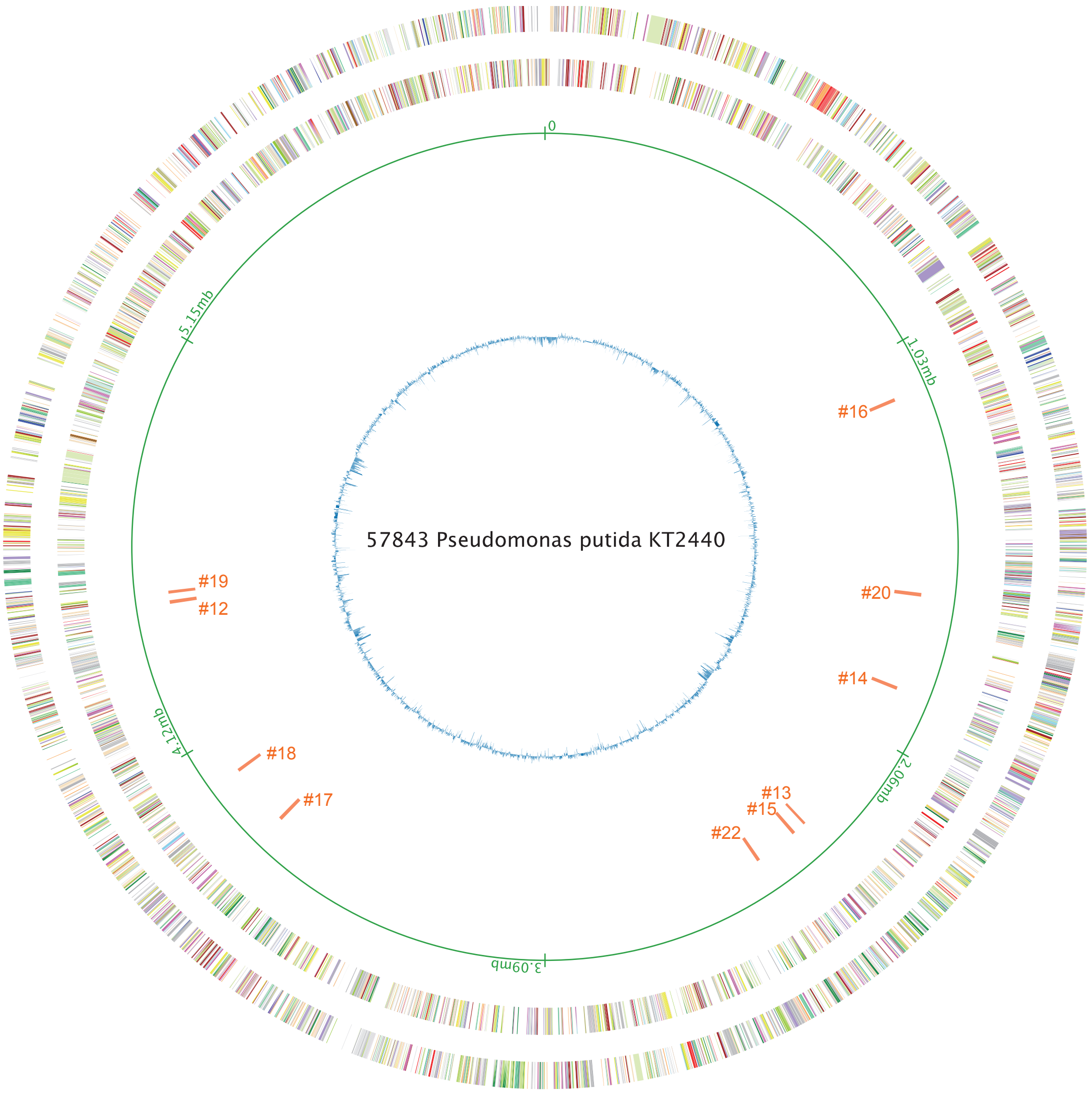

Figure S2 | Localization of *cscAB* integration along the chromosome of *P. putida*. Shown are (from outside to inside): Ring 1: OFRS plus strand (colored by COG category); Ring 2: OFRS minus strand (colored by COG category); Ring 3 (green): Coordinates; Ring 4 (orange): insertion sites of the *cscAB* construct; Ring 5 (blue): GC percentage. The numbers correspond to the clone numbers (table 2).

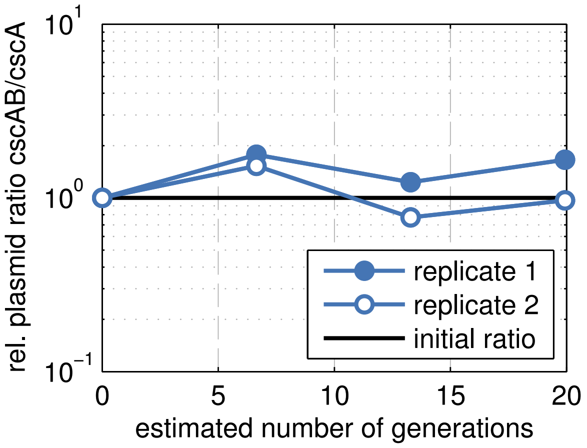

Figure S3 | Competition of *P. putida* (pSEVA224-*cscA*) and *P. putida* (pSEVA224-*cscAB*) during growth on sucrose. The ratio of both plasmids over time with respect to the initial ratio within the corresponding culture is depicted for two independent cultures. Stationary phase cultures of *P. putida* (pSEVA224-*cscA*) and *P. putida* (pSEVA224-*cscAB*) grown on M9 medium with 15.2 mM glucose were mixed in a volumetric ratio of 1:1, plasmids were isolated from this mixed culture directly after mixing to determine the initial relative abundances of each plasmid. The cultures were incubated at 30°C and an 10 µL aliquot was transferred every 24 h in fresh M9 medium with 16.7 mM sucrose. After each transfer, plasmids were isolated from the remaining culture with the NucleoSpin® plasmid Miniprep kit according to the suppliers manual for low copy plasmids. A 10 µL aliquot of the eluate was digested with EcoRI HF (New England BioLabs, NEB) to linearize the plasmids and the whole restriction mixture (20 µL) was loaded on a 1 % agarose gel prestained with Roti®-GelStain (Carl Roth). The intensity of the bands corresponding to each plasmid was determined with the ImageJ Gel Analysis tool and the ratio of the plasmid abundance was calibrated to the DNA ladder (NEB) used for the gel. Note that even after 4 transfers, which should reflect about 20 generations, neither of the both plasmids significantly prevailed over the other, which suggests that none of the strains had a detectable selective advantage over the other.

References

Boyer, H.W. and Roulland-Dussoix, D. (1969) A complementation analysis of the restriction and modification of DNA in *Escherichia coli*. *J Mol Biol* **41**: 459–472.

Grant, S.G., Jessee, J., Bloom, F.R., and Hanahan, D. (1990) Differential plasmid rescue from transgenic mouse DNAs into *Escherichia coli* methylation-restriction mutants. *Proc Natl Acad Sci U S A* **87**: 4645–4649.

Kessler, B., de Lorenzo, V., and Timmis, K.N. (1992) A general system to integrate *lacZ* fusions into the chromosomes of gram-negative eubacteria: regulation of the *Pm* promoter of the TOL plasmid studied with all controlling elements in monocopy. *Mol Gen Genet* **233**: 293–301.

Martínez-García, E., Aparicio, T., de Lorenzo, V., and Nikel, P.I. (2014) New transposon tools tailored for metabolic engineering of gram-negative microbial cell factories. *Front Bioeng Biotechnol* **2**: 46.

Pflüger-Grau, K., Chavarría, M., and de Lorenzo, V. (2011) The interplay of the $EIIA^{Ntr}$ component of the nitrogen-related phosphotransferase system (PTS^{Ntr}) of *Pseudomonas putida* with pyruvate dehydrogenase. *Biochim. Biophys. Acta* **1810**: 995–1005.

Silva-Rocha, R., Martínez-García, E., Calles, B., Chavarría, M., Arce-Rodríguez, A., Las Heras, de, A., et al. (2013) The Standard European Vector Architecture (SEVA): a coherent platform for the analysis and deployment of complex prokaryotic phenotypes. *Nucleic Acids Research* **41**: D666–75.

Waksman, S.A. and Reilly, H.C. (1945) Agar-Streak Method for Assaying Antibiotic Substances. *Ind. Eng. Chem. Anal. Ed.* **17**: 556–558.

Wilde, E. (1962) Untersuchungen über Wachstum und Speicherstoffsynthese von Hydrogenomonas. *Archiv. Mikrobiol.* **43**: 109–137.
